# Numerical analysis of permeate flux in reverse osmosis by varying strand geometry

**DOI:** 10.1038/s41598-022-20469-0

**Published:** 2022-10-05

**Authors:** Gohar Shoukat, Hassaan Idrees, Muhammad Sajid, Sara Ali, Yasar Ayaz, Raheel Nawaz, A. R. Ansari

**Affiliations:** 1 School of Mechanical and Manufacturing Engineering (SMME), National University of Sciences and Technology (NUST), Islamabad, 44000 Pakistan; 2 Artificial Intelligence for Mechanical Systems (AIMS) Lab, School of Interdisciplinary and Engineering Sciences (SINES), National University of Sciences and Technology (NUST), Islamabad, 44000 Pakistan; 3 National Center of Artificial Intelligence (NCAI), National University of Sciences and Technology (NUST), Islamabad, 44000 Pakistan; 4 Staffordshire University, Stoke-On-Trent, ST4 2DE UK; 5 Department of Mathematics and Natural Sciences, Centre for Applied Mathematics and Bioinformatics, Gulf University for Science and Technology, 73F2+GV4 Mubarak Al-Abdullah, Kuwait; 6 Unit A2, Nutgrove Office Park, Rathfarnham, Gavin and Doherty Geosolutions (GDG), D14 X627 Dublin, Ireland; 7 Human-Robot Interaction (HRI) Lab, School of Interdisciplinary Engineering & Science (SINES), National University of Sciences and Technology (NUST), 44000 Islamabad, Pakistan

**Keywords:** Engineering, Mechanical engineering

## Abstract

In regions with limited potable water availability, membrane desalination is being employed to filter water using a pressure-driven approach. Because of the high energy consumption required to produce the pressure differential needed for this method, researchers have been trying different geometric designs of spacer filaments to enhance the amount of permeate flux in terms of energy utilization. The purpose of spacer filaments is to support membranes structurally and induce turbulent mixing in spiral wound membrane desalination. In this paper, the improvement of mass transfer in desalination driven by reverse osmosis has been studied using Computational Fluid Dynamics (CFD) with the introduction of spiral wound membranes that are lined with spacer filaments in a zig-zag formation having alternating diameters for strands. The fluid flow characteristics for a 2-dimensional geometric model were resolved using the open-source program OpenFOAM by changing the Reynolds number to just before the inception of instabilities. Ratios of alternate strand diameters were also varied between one and two. Based on a detailed analysis of velocity contours, pressure distribution, wall shear stresses, and steady-state vortex systems, the research findings offer guidance for employing alternating strand design in zig-zag formation for optimum mass transfer and minimal pressure drop when accounting for concentration polarization.

## Introduction

Recent years have seen Reverse Osmosis (RO) desalination technique gain a wider market share for water purification because of technological development and a large quantity of seawater^1–3^. Fluid with dissolved salts is pumped at high pressures across a hydrophilic membrane that separates the fluid from its impurities. The concentrated fluid is held in the retentate channel while the fluid is passed through the membrane into the permeate channel by the membrane^4^. The type of material used also affects the permeability. Nanomaterials allow greater permeability when compared to other materials^5^. However, the exceptionally elevated pressures lead to remarkably high operational costs. The considerable energy utilization of the membrane desalination process for water purification has persuaded researchers to investigate different spacer designs using computational techniques for minimizing energy consumption per unit of permeate^6–8^. The desalination processes are also of economic benefits minimalizing the feed and brine volumes^9^. Spiral wound membrane (SWM) is widely used amongst the many forms of membrane modules in the industry compared to rectangular channels due to its high density of membrane packing. Further demands on the RO process, including a reduction in operating cost, extension in the life of the membrane, and enhancing membrane module and system designs, can be addressed by incorporating functionalized feed spacers. They also have an additional advantage in household purification, where the water recovery rate is very high^10–14^. SWM has spacer nets along their entire length that serve two functions: (1) they offer mechanical support to multi-layered permeate and retentate channels, and (2) they induce instabilities in the flow regime.

Reverse osmosis is primarily impacted by Concentration Polarization, as stated by Yun et al.^15^, Bahmanyar et al.^16^, and Benjamin et al.^17^. This phenomenon can be explained by the variance in concentration along with the height of the channel, with the walls being the most saturated with salt. Salt along the channel continues to build up with time as water carrying this salt permeates through to the other side of the membrane, leaving behind its load, which sediments along with the layer of the membrane. This layer of salt adds to the resistance to water permeation by rendering that specific area practically impermeable, thereby reducing the effective surface area for the exchange of fluid to occur. The saline wastewater from the agricultural and industrial activities is a source of high salt concentration and organic content including other pollutants harming the environment^18^. Reverse Osmosis is equally effective for extracting the salt from the wastewater obtained from the agricultural and industrial sources. The concentration polarization in reverse osmosis reduces the effective osmotic pressure difference across the membrane active layer^19^. In comparison of osmotic pressure to various types of concentration polarizations, the Osmotic pressure proportion for dilutive concentration polarization increases while it tends to decrease for concentrative concentration polarization^20^. Ochando et al.^21^ demonstrated that permeate flux reduced by around 39% due to salt deposited along the membrane. Christopher et al.^22^ concluded a similar decrease in permeate flux due to concentration polarization. Zhou et al.^23^ explored whether the mixing and mass transfer are enhanced for laminar flow conditions in reverse osmosis (RO) membranes. It was found that for patterned membranes, the nominal permeate flux increases up to 40% compared to flat membranes.

Hasan et al.^24^ introduced a new feed spacer geometry for the Forward Osmosis (FO) to achieve more water flux than the conventional models. The models presented in the research achieved 8% more water flux when compared to commercial models. The efficiency of an SWM is limited by membrane biofouling. Lin et al.^25^ used 16 feed spacers with varied geometries and channel porosities for biofouling experiments. The results showed that the anti-biofouling and hydraulic performance were more responsive to the diameter variation and spacer thickness. Diaz et al.^26^ set up a nanocomposite-based RO module and evaluated their fouling resistance in seawater desalination conditions. It was found that the RO modules were ideal for desalination plants as the required operational maintenance was less as compared to the commercial membranes. But the RO-based plants can be affected by the presence of “protobiofilms,” which results in membrane biofouling of such plants, as discussed by Winters et al.^27^. This can be reduced by decreasing the flow velocity into the Rotary Energy Recover Devices (RERD), which slows down the rate of biofilm formation and rate of RERD biofilm dispersal.

Turbulence promoters can be effectively utilized to reduce concentration polarization by periodically disturbing the boundary layer^28,29^. Geraldes et al.^30^ initiated the use of such turbulence promoters and used ladder-type arrangement of spacers in the feed channel. His investigation yielded significant gains in permeate flux. He also highlighted that although the pressure drops across the channel increased, the gain in permeate flux outweighed the enhanced energy requirements. Da Costa et al.^31^ and Thomas et al.^32^ also shared their conclusions. Their studies identified more significant mass transfer due to increased unsteadiness within the feed channel. Subramani et al.^33^ studied shear rates and corresponding pressure drops in open channels and spacer-filled channels and emphasized the benefits of channels filled with spacers. Santos et al.^34^ developed on the work of Geraldes et al.^30^ and identified the most efficient designs of spacers for spiral wound membrane modules. They experimented with twelve cross-sections of ladder-type modules, including but not limited to rectangular and circular types, and reported on the transition from laminar to turbulent. Da Costa et al.^31^ also identified that the zig-zag spacer configuration with a circular cross-section was the most efficient of the configurations he experimented with. Gu et al.^35^ find new dimensionless correlations for friction factor and concentration polarization using CFD simulations, which can be used to permeate flux on CP without having recourse to the film theory. Shoukat et al.^36^ studied zig-zag spacer design with elliptical cross-section filaments, with flow having 45 degrees orientation in the 3D spacer unit direction. It was concluded that if the elliptical projection of the circular domain was ignored, the margin in error for the mass transfer coefficient would increase significantly.

The configurations of spacers being used for RO spiral wound elements were developed using experimental studies^37^ and hydraulic modeling, leading to a bi-planar net configuration with square or rhomboid openings. Kavianipour et al.^12^ performed a 3-dimensional steady-state CFD analysis to compare Ladder-type, Triple, Wavy, and Submerged feed spacer configurations, finding that the Ladder-type was better for Re < 120 while Wavy was better for Re > 120. Ling et al^38,39^ developed a solver in OpenFOAM to solve a transient Navier–Stokes Equation with advection–diffusion and adsorption–desorption equations for foulant accumulations. The model was applied to membranes with topological and morphological alterations, i.e., membranes with wiggly shapes and flat-rectangular reference cases. The model when applied at the membrane boundary were able to predict the macroscopic properties such as permeate flux, pressure drop with a good match with the experimental results. Normal strands which have the same diameters are extensively utilized. The struggle to achieve greater permeate flux at a lesser cost requires enhanced designs of strands. Tielen^40^ and Atkinson^41^ report on experimental and computational studies of feed spacers with three different strand types: equal, alternating, and bottleneck. These were aimed at decreasing pressure drop and minimizing low flow areas. The results showed that the alternating strand type feed spacer is better in terms of pressure difference while reducing areas of low feed water velocity. Bin et al.^42^ used CFD software to study five different kinds of capillaries to simulate their parameters. The results showed that increasing the super-cooling effect of a liquid can increase the liquid volume fraction in capillaries. Besides these publications, alternating strand design has received limited attention in recent literature.

This work focuses on the novelty of Alternating Strand Diameters (ASD) in spacers using numerical analysis by exploring the performance parameters for various feed spacer strand size ratios in ASD geometric designs. Since the permeate flux and concentration polarization are coupled, we employed a decoupled CFD approach allowing us to study the two phenomena independently. The permeate flux is affected by the concentration buildup along the walls of the membrane; as the layer of the salt deposited builds up, the incoming flow of water meets added resistance to permeate through the membrane into the permeate channel. Thus, the concentration polarization retards the performance significantly, and better estimation of the filtration ability of a membrane requires numerically decoupling these two aspects. In this study, we focused on concentration polarization and membrane hydrodynamics separately. The latter primarily determines the maximum permeate flux per pressure drop unit across the unit cell. The decoupled flow prevents concentration polarization from impacting the unit’s performance, allowing it to operate at its full potential and serving as an ideal case. The spacer geometry modification increases the efficiency of a spiral wounded membrane^43^.

Three-dimensional modeling of spacer strands proves to be computationally intensive. Iwatsue et al.^44^ investigated flow inside a cubic-driven cavity and concluded that steady-state flow at lower Reynolds Numbers could be modeled in two spatial dimensions without influencing the degree of accuracy of results too much. However, as Reynolds numbers increases, the end wall effects start to take over, which cannot be captured within the two dimensions, and hence a two-dimensional study is rendered useless. Our studies, therefore, discuss the results of simulations at low Reynolds Numbers.

The SWM, as the name suggests, is constituted by spiral channels. Running simulations on the domain as a straight channel still yields accurate results. The dimensioning of a section of the SWM feed channel is obtained from the findings of Li et al.^45^. To further ensure the accuracy of simulating the SWM section as a straight narrow channel instead of a curvilinear channel, Ranade and Kumar^46^ compared the results of fluid flow in a curvilinear channel with a narrow channel. Their findings were reiterated by Schock and Miquel^47^, who reported that flow in straight narrow channels is not significantly different from that in a curved channel.

## Methodology

### Computational domain

**Figure 1:**
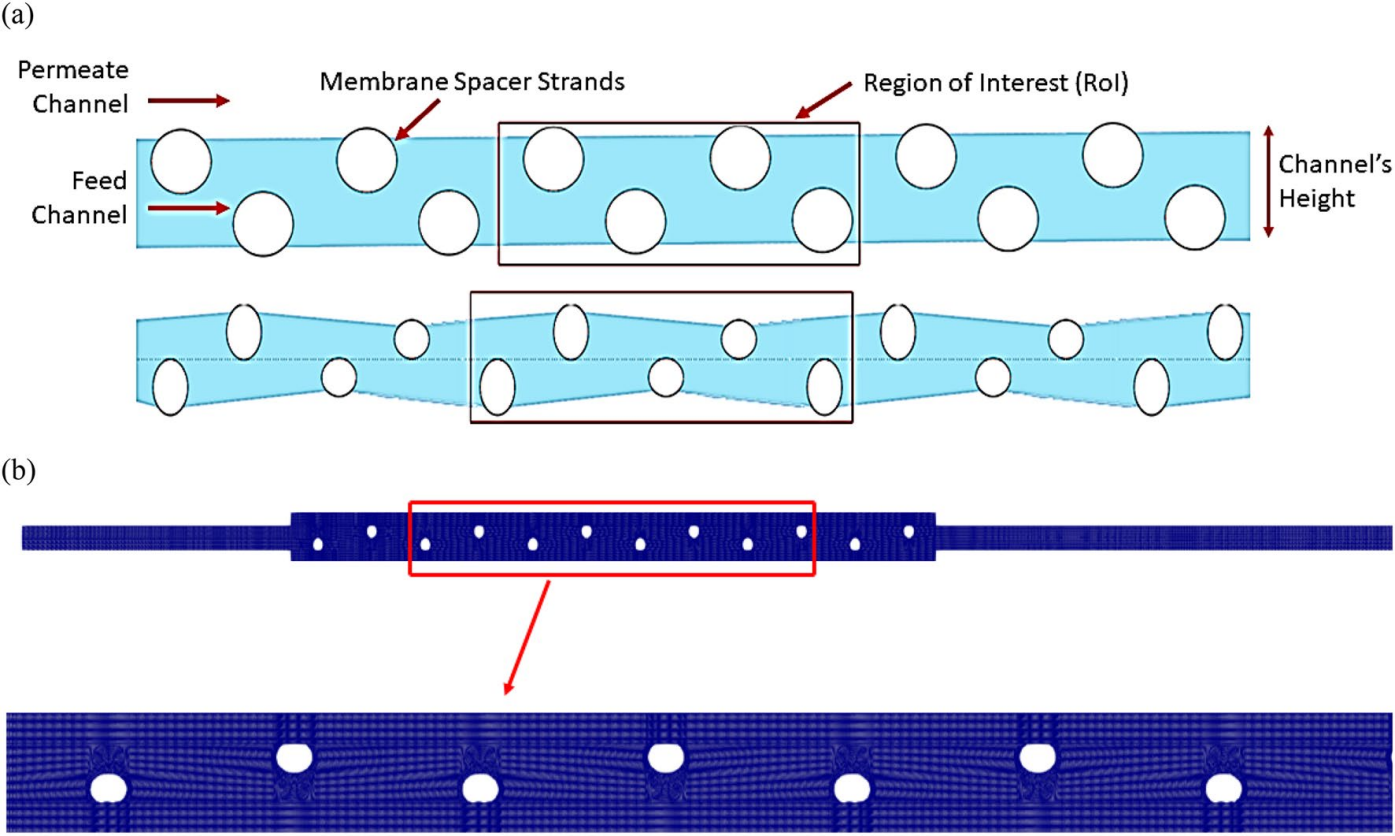
Computational domain (**a**) with strand ratio, R of ‘1’(above) and R of ‘2’ (below), region of interest (RoI) is highlighted in red (**b**) closeup view of mesh.

Santos et al.^34^ concluded that the Computational domain can be limited to consecutive longitudinal filaments to run a CFD analysis on a spiral wound membrane feed channel. Thereby limiting the computational resources required to run the simulation. Based on the repetitive symmetry of the physical domain, the numerical simulation was limited to consecutive longitudinal filaments. Three units of the Region of Interest (RoI) shown in Fig. 1 were modeled sequentially, using three-strand diameter ratios for type A analyses as detailed in Table 1. The strand ratio of 1:1 corresponds to standard case, while ratios of 1:1.5 and 1:2 are chosen to study potential improvements in performance parameters of spacers with Alternating Strand Diameters (ASD). The simulations were carried out at Reynolds number of 150 and 200, and the performance parameters were extracted from the second unit. This was added to ensure that the flow from the intake is fully developed and to prevent the exit boundary condition from affecting the region of interest.

**Table 1:** Summary of studies performed.

Purpose	Strand ratios
Concentration polarization analyses (type A)	1, 1.5, and 2
Hydrodynamic analyses (type B)	1, 1.5, and 2

The domain was constructed using the BlockMesh utility of OpenFOAM, an open-source library for computational fluid dynamics. The structured domain allowed for fine meshing near the membranes, where the bulk of the simulations focused on capturing the permeate flux through the membrane. To standardize the simulations, 124,320 cell nodes were kept. Between cases, the average non-orthogonality varied from 8.54 to 14.38. The residuals were controlled to stay under 10^–6^ for all properties except velocity, for which they were kept under 10^–5^. To ensure fair comparison the number of mesh elements were kept same in each case. The fluid within the domain is modeled as Newtonian with a kinematic viscosity of 10^–6^ (m^2^/s) at a temperature of 20 °C. Density changes and gravitational effects are insignificant and are therefore modeled as constants.

### Mathematical description

The mathematical description of the steady state, laminar flow of water over spacers placed between permeable membrane layers in reverse osmosis consists of the equations of conservation of mass Eq. (1) and momentum Eq. (2) coupled with convective transport equation Eq. (3) for concentration. The equations and their dependent variables are described below.

1 $$\nabla . \overrightarrow{\upsilon }=0$$

2 $$\nabla . \left( \overrightarrow{\upsilon } \overrightarrow{\upsilon } \right)- \nabla . \left(\upnu \nabla \overrightarrow{\upsilon }\right)= \frac{1}{\uprho }\nabla \mathrm{p}$$

3 $$\nabla .\left(C\right)- {D}_{c}{\nabla }^{2}C=0$$

where, $\overrightarrow{\upsilon }$ is the velocity vector (${\text{m}}/{\text{s}}$), $\upnu$ is the kinematic viscosity (${\text{m}}^{2}/{\text{s}}$), $\uprho$ is the fluid density (${\text{kg}}/{\text{m}}^{3}$), $\mathrm{p}$ is the density normalized pressure (${\text{m}}^{2}/{\text{s}}^{2}$), $C$ is the concentration of salts Transported Scalar (${\text{mol}}/{\text{m}}^{3}$)and ${D}_{c}$ is the concentration diffusion coefficient (${\text{m}}^{2}/{\text{s}}$).

**Figure 2:**
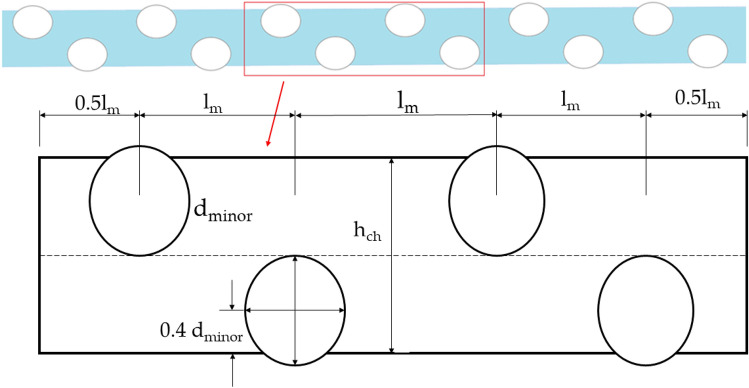
Detailed computational domain of the RoI for strand ratio, R of ‘1’. Where, the channel height, *h*_*ch*_ is 0.00364 m and *d*_*minor*_ is 0.0026 m.

The channel's height is considered the characteristic length and employed in estimating the Reynolds number, as shown in Fig. 2. Channel Reynolds Number, *Re*_*ch*_ was used, which is calculated using channel height, *h*_*ch*_ as follows:

4 $${\mathrm{Re}}_{\mathrm{ch}}= \frac{\upupsilon {\mathrm{h}}_{\mathrm{ch}}}{\upnu }$$

For the second case, membrane hydrodynamics were incorporated by attenuating the time derivative to the above equations and introducing a source term *S*_*i*_ in the momentum equation. The source term, *S*_*i*_, is composed of two parts, a viscous loss term, $\mu D$ and an inertial loss term $\frac{1}{2}\rho \left|{u}_{ij}\right|F$, creating a pressure drop proportional to the velocity and velocity squared. The following Darcy-Forchheimer equation defines it.

5 $${\mathrm{S}}_{i}= -\left(\mu D+\frac{1}{2}\rho \left|{u}_{ij}\right|F\right)$$

A constant velocity boundary condition was used at the inlet boundary, while a zero-velocity gradient was imposed at the outlet boundary. No-slip on the wall patches and zero gradients on the outlet were used as velocity boundary conditions. Constant zero-gauge pressure was defined at the outlet. There is flow across the membrane from the feed channel to the permeate channel. Concentration was defined to be 0.006 mol/m^3^ at the inlet and Neumann type at the walls and the outlet. Structured meshing was carried out using the block Mesh utility of OpenFoam to describe the spacer curvatures, and the numerical simulations that were carried out in OpenFOAM^48^. The simpleFoam and porousSimpleFoam solvers were employed for the two cases, respectively. The divergence terms were discretized with Gauss upwind numerical scheme while Gauss linear scheme was used for all other terms.

## Results and discussions

**Figure 3:**
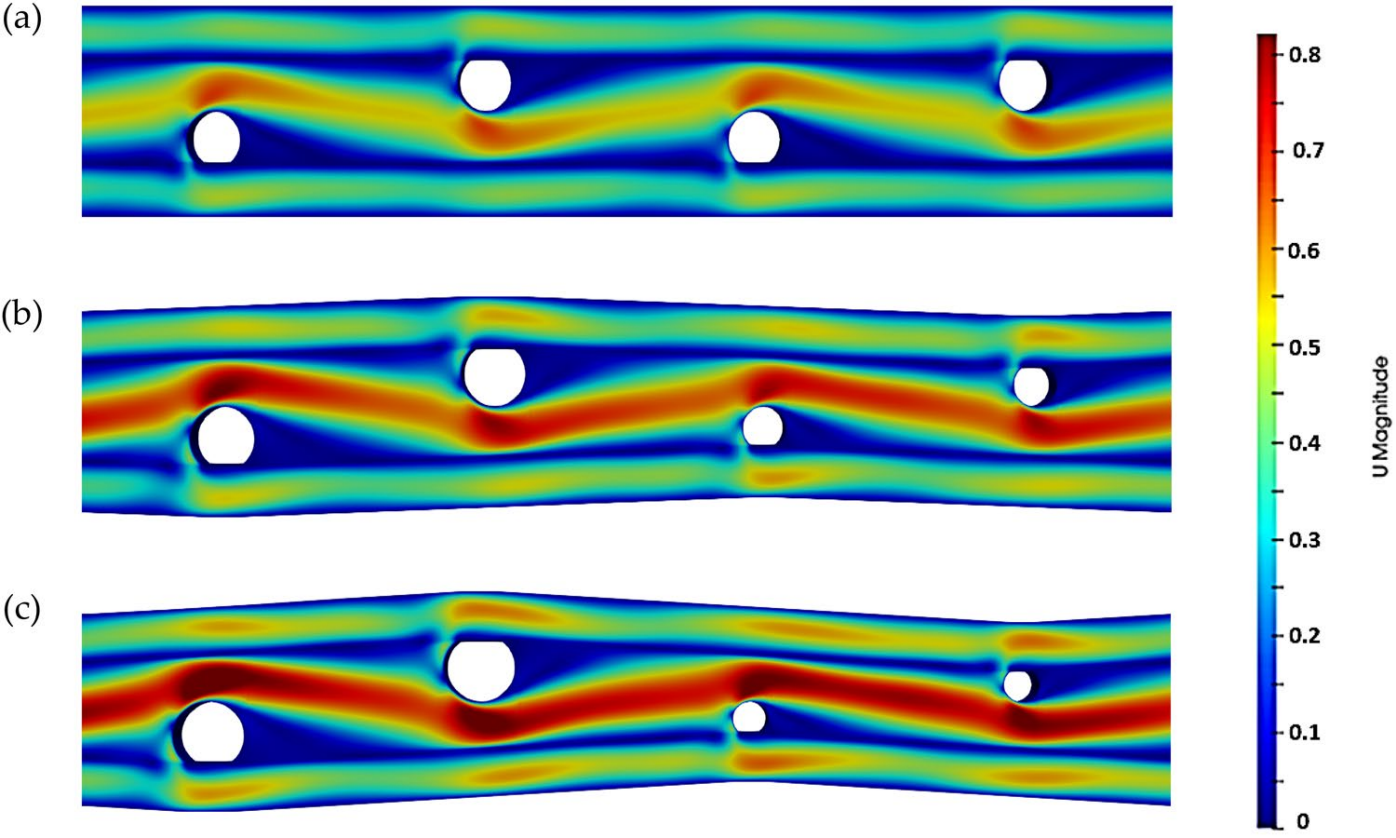
Velocity field with a strand diameter ratio of (**a**) R of ‘1’, (**b**) R of ‘1.5’and (**c**) R of ‘2’.

The velocity field distribution obtained from numerical simulation is depicted in Fig. 3 for alternating strands with the same diameters (top) and alternating strands with a diameter ratio of 2 (bottom). The latter image shows the relatively higher velocities achieved with the same input velocities, which contribute to higher local Reynolds Numbers leading to more significant vorticity and turbulence.

The periodically occurring converging channels assist in speeding up the fluid. When this fluid encounters the membrane walls, it rapidly decelerates, resulting in high stagnation pressures, which outweigh the pressure on the permeate side of the membrane, thereby encouraging fluid migration. The impact of higher trans-membrane pressure leading to a more significant pressure drop can be more clearly observed near the front of the strands, where there is a marked increase in fluid transfer.

Permeate flux is affected by the concentration buildup along the walls of the membrane. As the layer of the deposited salt builds up, the incoming flow of water meets added resistance to permeate through the membrane into the permeate channel. A static pressure differential between the retentate and permeate channels is required to force the water through. The layer of salt deposited along the walls necessitates a more significant pressure differential to achieve the same amount of permeate flux. By reducing the strand diameter of the alternate strand, the impact of the turbulence promoters is further enhanced as the converging–diverging channel adds to the vorticity. This is exceptionally useful in reducing the concentration polarization. The swirling flow of water scrapes off the deposited salt from along the membrane and thrusts it back into the water flow, enhancing mixing.

### Concentration polarization and membrane hydrodynamics for R1

**Figure 4:**
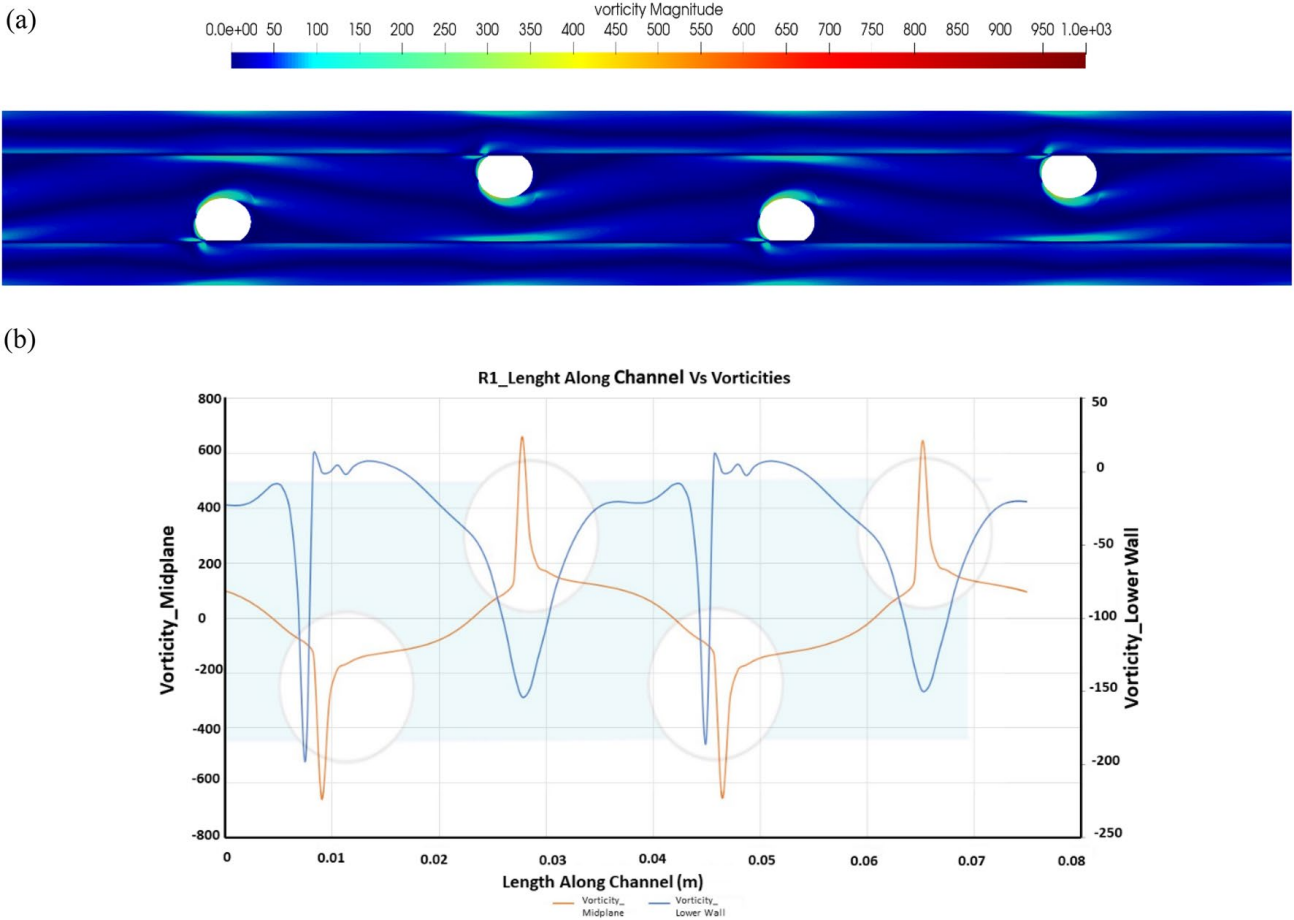
Vorticity plot for R1 (**a**) vorticity distribution along the channel and (**b**) center line vorticity vs. vorticity along the lower wall of the channel.

The vorticity ω = ∇>$\overrightarrow{\upsilon }$ is computed as the curl of the velocity vector along the length of the channel at the mid-plane of the domain for the idealized decoupled computation is shown in Fig. 4. As a comparison for the Re200, the peak vorticity is recorded to be 659. The increased vorticity can be attributed to the curving channel, as shown by the figure above.

**Figure 5:**
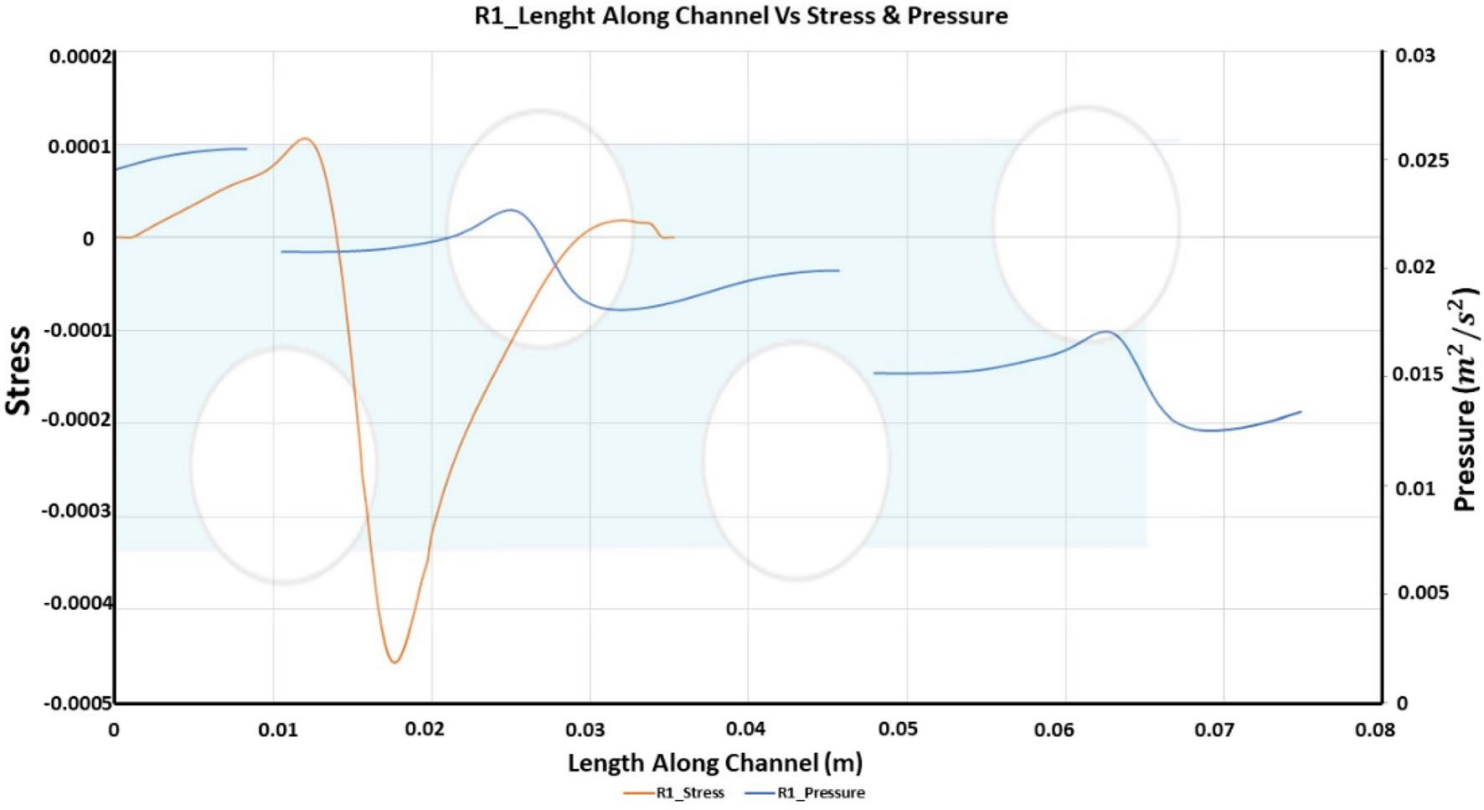
Length along channel vs stress and pressure for R1.

Wall Shear Stresses elevates the pressure drop and reduce the concentration polarization^49,50^. These stresses could wear away salts deposited on the membrane, subsequently improving permeate flux. A bigger separation bubble leads to greater recirculation and mixing. To determine the extent of recirculation, function at the bottom wall was recorded and is shown in Fig. 5. The wall shear stress calculated as τ = R⋅n, where τ is the wall shear stress, R is the shear stress symmetric tensor and n is the patch normal vector.

The channel length varies with the separation bubble's length as well. Therefore, the lengths of the wake region are normalized over the channel length for comparison. If we also consider the pressure drop across the channel in Fig. 5, it shows the normalized density pressure at the bottom wall along the length of the channel.

**Figure 6:**
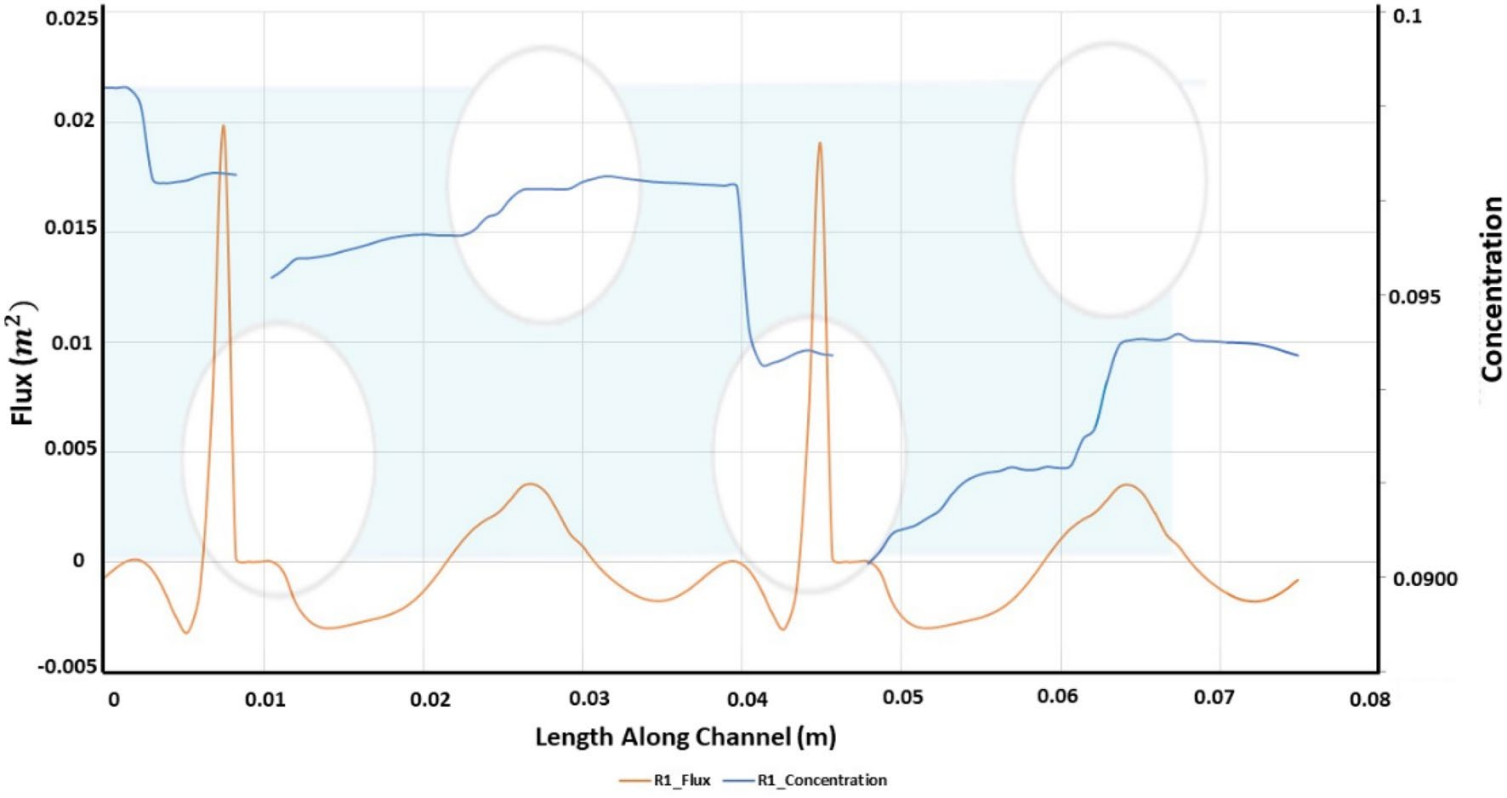
Length along channel vs flux and concentration.

The added resistance to permeation due to salt buildup along the membrane wall has already been established. Figure 6 illustrates how effectively a channel lined with Alternating Strand Diameter (ASD) combats polarization. The graph also shows a series of periodic rises and declines in concentration. The steepest declines occurred in the region within the domain where the constriction in diameter begins leading to greater unsteadiness within the fluid field and hence, lower concentration polarization. Figure 6 shows a series of ascent and descent in the concentration with the steepest decline occurring at the region where constriction in the diameter begins coupled with an increase in the osmotic pressure.

For comparison of membrane hydrodynamics along channel length, the channel is sandwiched among layers of porous membranes. This allows analysis of permeate flux and flows characteristics such as vorticity and pressure drop for ultimate comparison with the idealized case previously presented. The permeate flux through the lower wall for R1 is shown in Fig. 6.

### Concentration polarization and membrane hydrodynamics for R1.5

**Figure 7:**
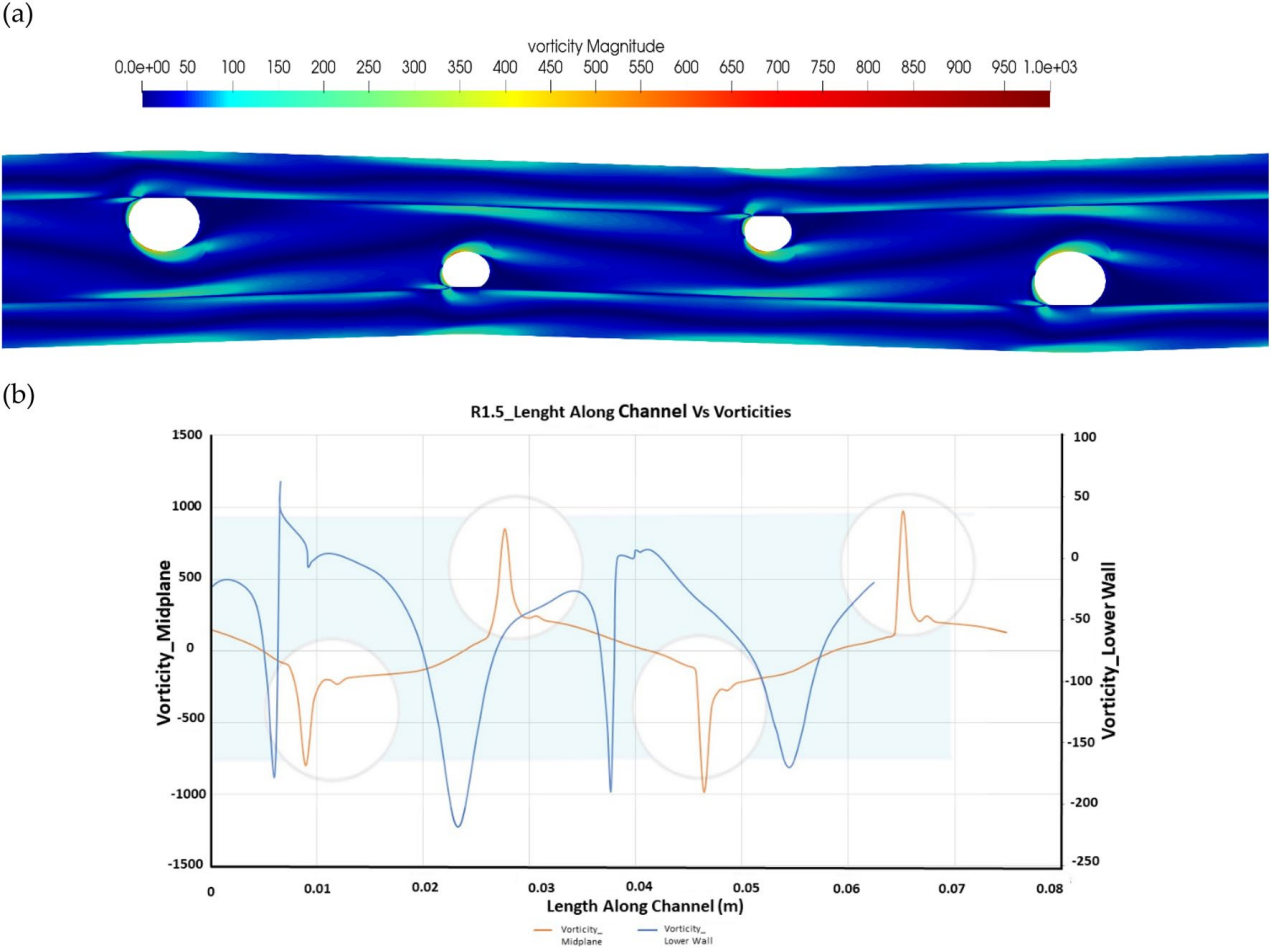
Vorticity plot for R1.5 (**a**) vorticity distribution along the channel and (**b**) Center line Vorticity Vs. vorticity along the lower wall of the channel.

For a strand ratio of R1.5 along the channel length, the maximum vorticity was recorded to be 801. The center line vorticity is observed to be increasing along the length of the channel while decreasing on the bottom line of the channel. The vorticity trends for strand ratio 1.5 are shown in Fig. 7.

**Figure 8:**
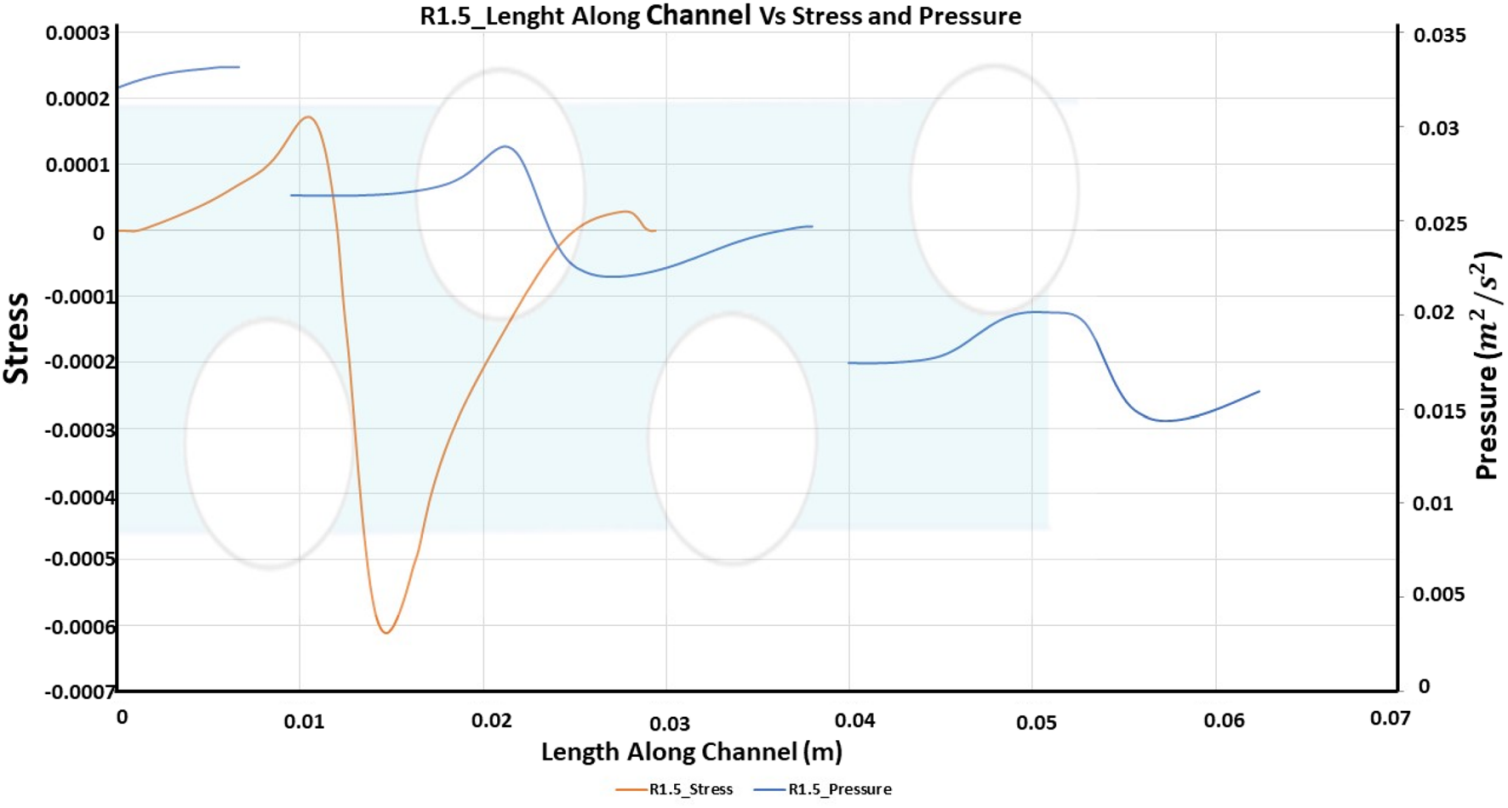
Length along channel vs stress and pressure for R1.5.

Figure 8 shows the wall shear stress and normalized pressure at the bottom wall across the channel for a diameter ratio of 1.5. This represents the dependence of channel length variety on the separation bubble. Therefore, the lengths of the wake region are normalized over the channel length for comparison. The trend is the same as that for R1. Local pressure accompanied by stress along channel length is depicted for R1.5. The local pressure obtained is greater than R1, which shows that the trend increases for higher strand ratios.

**Figure 9:**
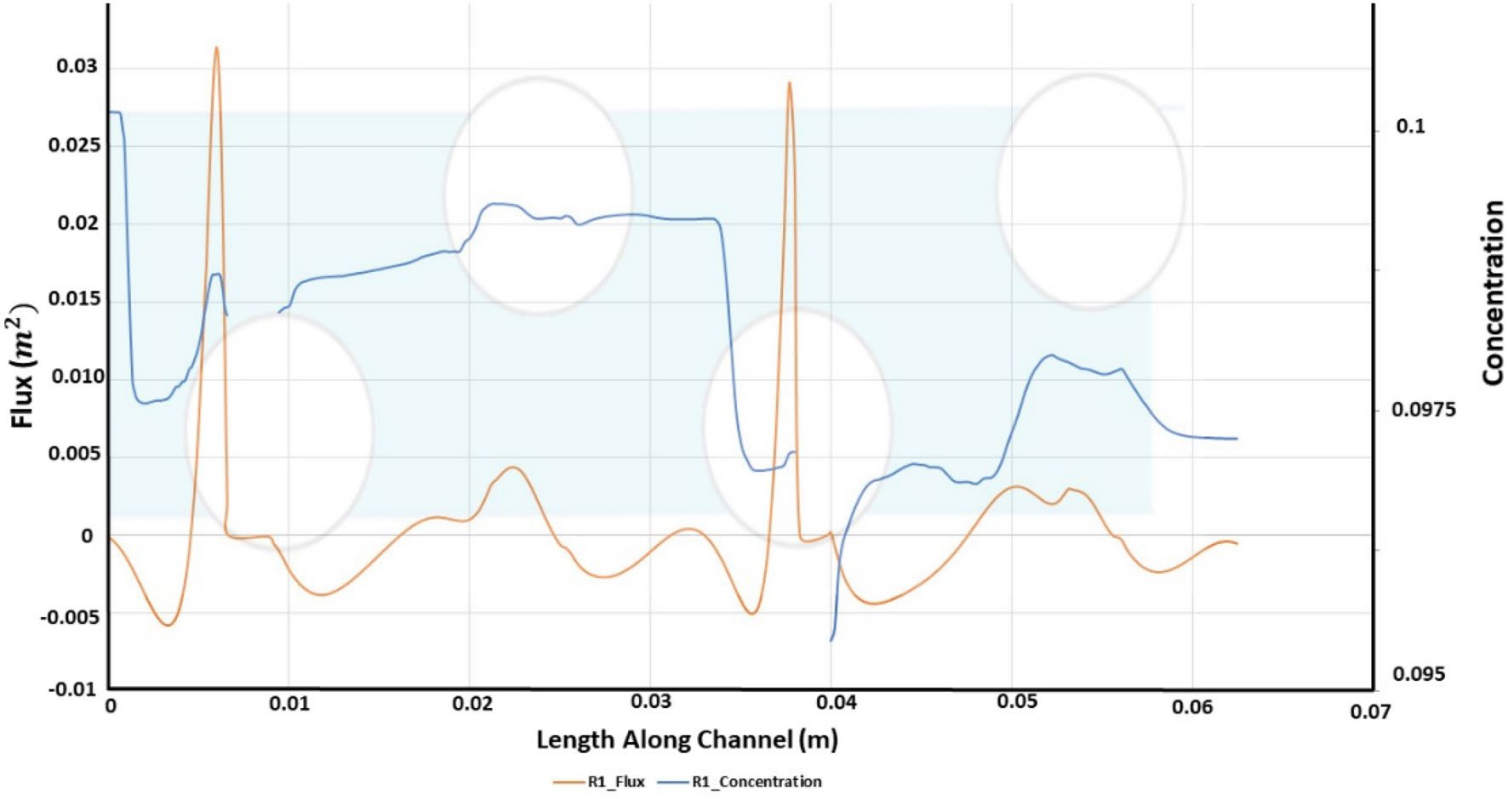
Length along channel vs flux and concentration for R1.5.

In Fig. 9, the concentration and flux are plotted along the length of the channel. The graph depicts that the concentration has a periodic rise and decline for the channel length. The lower concentration polarization is obtained in the region where the domain constriction begins. The regions with increase concentration polarization experiences reduced Osmotic pressures and vice versa in location where cross-sectional area expands. In comparing flux with the concentration, greater flux is received in the region of high-pressure drop. However, the flux for R1.5 is greater than that observed for strand ratio R1.

### Concentration polarization and membrane hydrodynamics for R2

**Figure 10:**
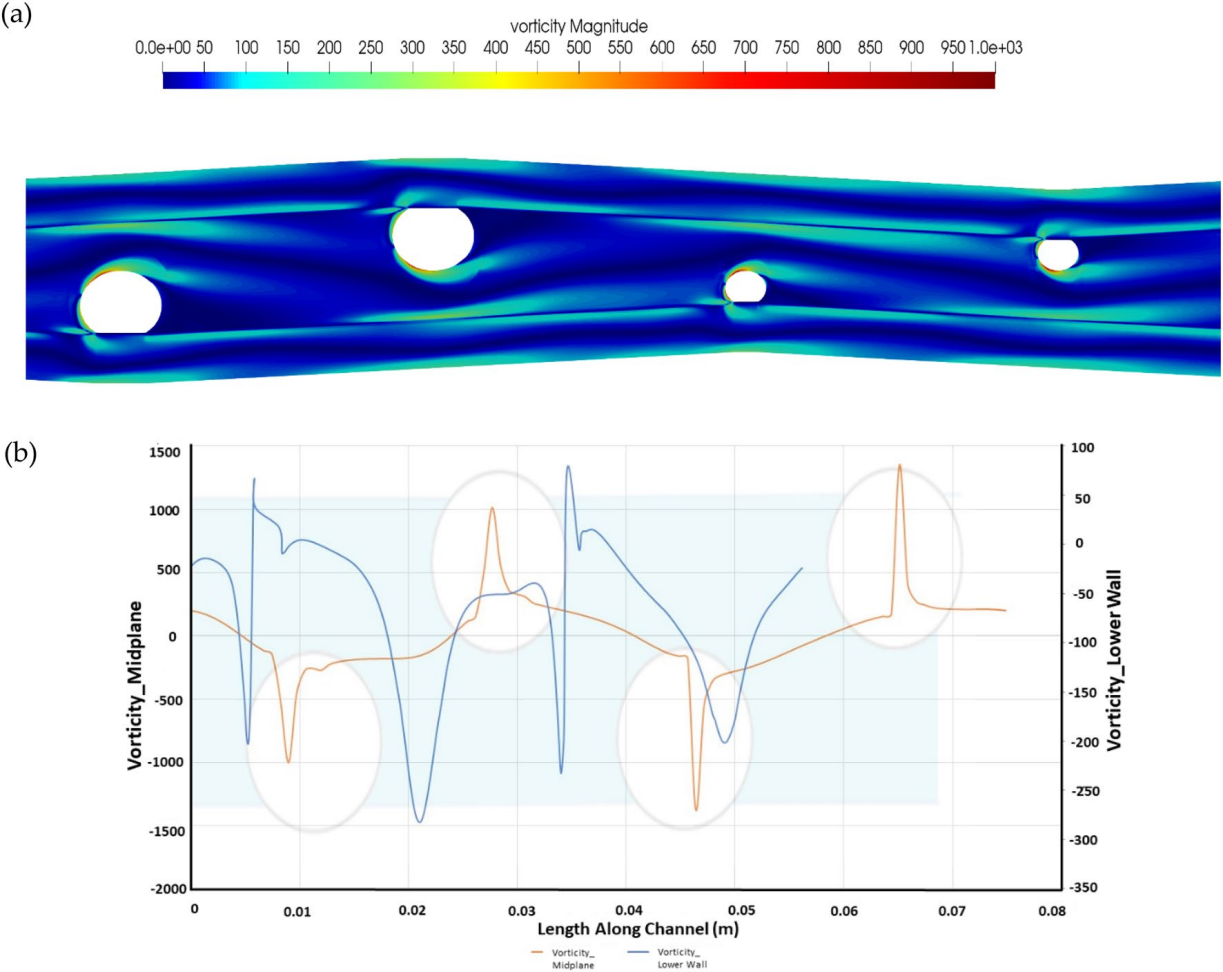
Vorticity plot for R2 (**a**) vorticity distribution along the channel and (**b**) center line vorticity vs. vorticity along the lower wall of the channel.

The graph of vorticity along the channel length for strand ratio R2 is given in Fig. 10. The vorticities for R2 progressively increase, with a maximum value of 1009, along the channel length as compared to R1 and R1.5. The increasing trend can be attributed to the geometry, i.e., the curving channel.

**Figure 11:**
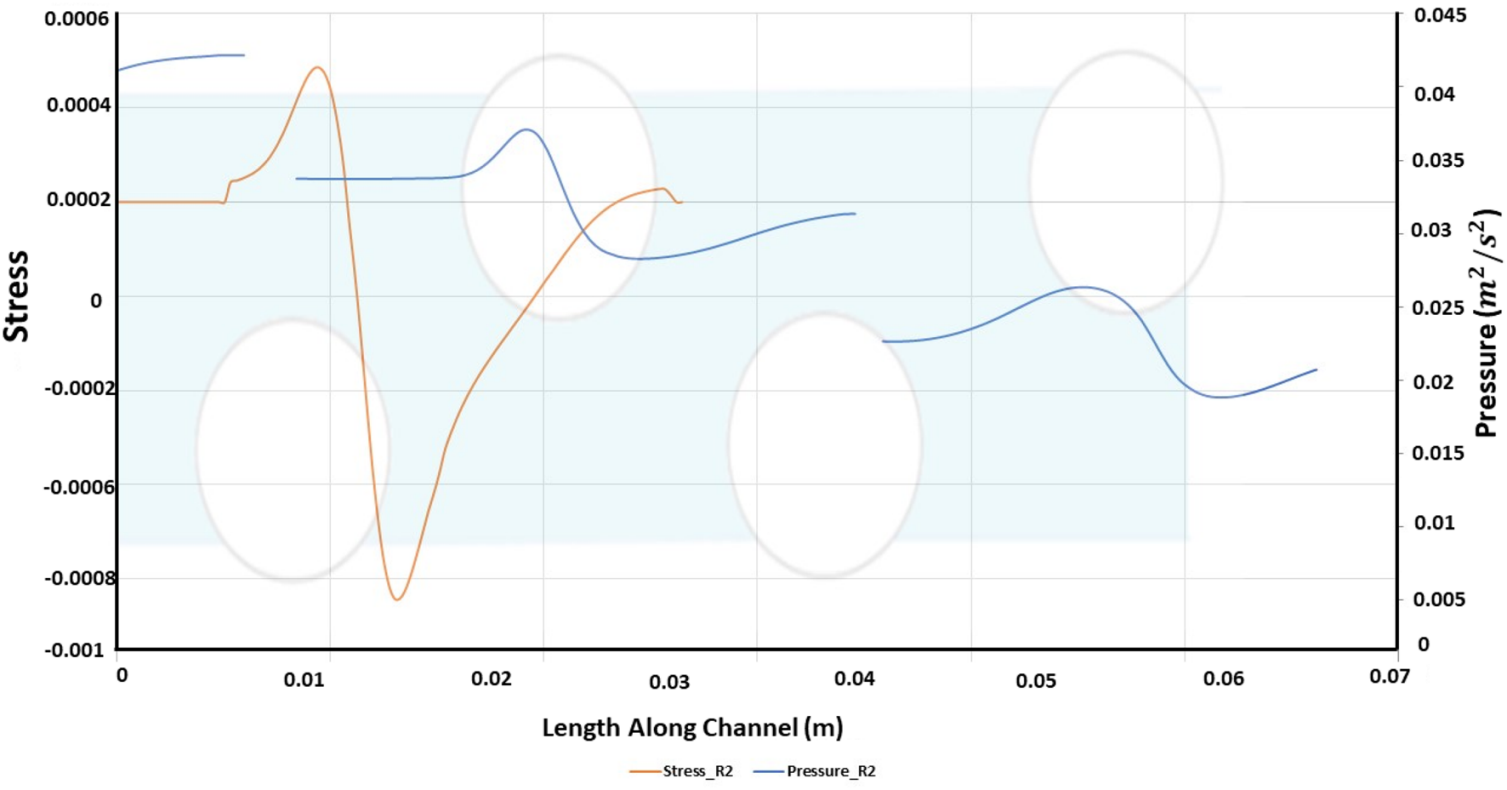
Length along channel vs stress and pressure for R2.

The wall shear stress and normalized pressure at the bottom wall of the membrane for a strand ratio of 2 have the same trend as that of R1 and R1.5, shown in Fig. 11. The wake region length is normalized along the length of the channel. The local pressure trend keeps increasing as the strand ratio increases, which means that the local pressure for R2 is greater than that of R1 and R1.5. The increase in the local pressure is obtained due to the area constriction region caused by convergence and divergence. Local pressure for R2 is greater when compared to other strand ratios.

**Figure 12:**
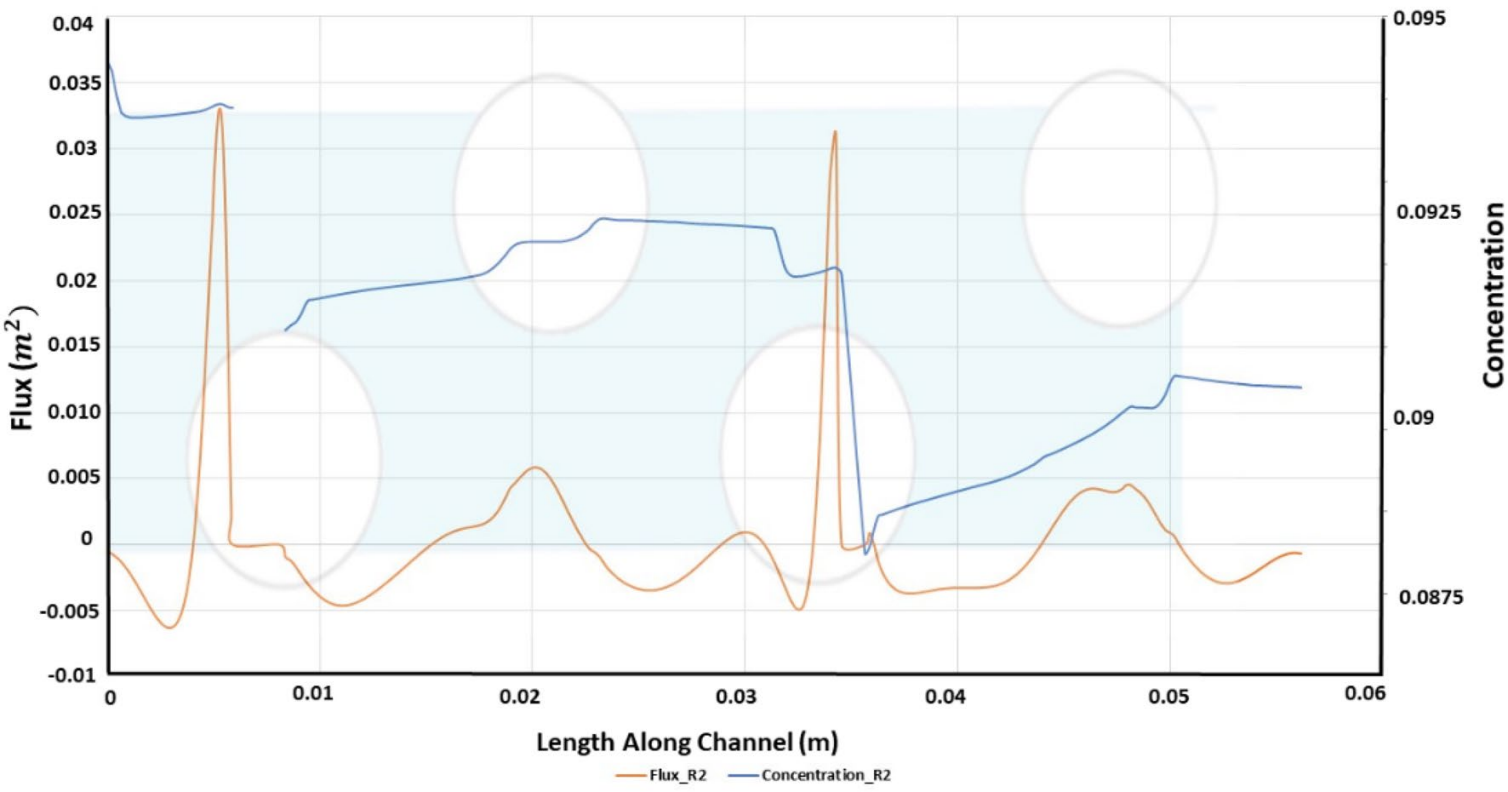
Length along channel vs flux and concentration for R2.

The concentration and flux along the length of the channel for strand ratio R2 is given in Fig. 12. The trends obtained for both concentration and flux along the channel in like strand ratio of 1 and 1.5 but a decline observed for the later cases, i.e., for R1.5 and 2. This is because for Ratio 1, the salt builds up rapidly along the membrane while there is a decelerated buildup observed for R1.5 and R2. On the other hand, permeate flux is greater for R2 due to a higher pressure drop. The flux increase also affects the vorticity, which for the same strand ratios is on the higher side. As The decline of concentration polarization occurs more for strand ratio R2 due to rapid building of salts along the membrane, the osmotic pressure has a greater increase observed than the other strand ratios.

Comparing Fig. 6, 9 and 12, as strand ratio is increased, permeate flux increases and concentration polarization decreases, while Pressure Drop, Stress and Vorticity increase. The improved performance of ASD can be explained by increasing vorticity that minimizes concentration on the lower surface. The cost of this advantage is represented by increased pressure drop across the channel.

### Static pressure comparison for all strand ratios

The local pressure is observed to be increasing for higher strand ratios. A greater increase in static pressure is observed due to the area constriction caused by the convergence and divergence of the channel cross-section. A permeate flux relation was described by Kedem and Katchalsky^51^ and Merten^52^ which further highlights the importance of a higher local pressure to obtain greater permeation through the membrane.

6 $$J={L}_{p}\left(\Delta {p}_{tm}-\sigma {\Delta \pi }_{tm}\right)$$

where, J is the flux of solution through the membrane (${\text{kg}}/({\text{m}}^{2} {\text{s}})$), *L*_*p*_ is Membrane hydraulic permeability coefficient ${\text{kg}}/({\text{Pa}} {\text{m}}^{2} {\text{s}})$, $\Delta {p}_{tm}$ is the transmembrane pressure drop (Pa), $\sigma$ is the reflection coefficient, and ${\Delta \pi }_{tm}$ is Transmembrane osmotic pressure drop (Pa). The evolution of the pressure drop across the channel with an increase in alternating strand ratio highlights the increase in operational cost with this spacer design. The benefits of this approach to the filtration of minerals from the feed water will be examined in the next section.

### Flux comparison for all strand ratios

R2, as predicted, generates greater flux, albeit at a much higher pressure drop, to achieve a relatively little difference in permeate flux. As expected, the higher ratios instigate greater vorticity in the channel, as shown in Fig. 10. While this is considerably smaller than the idealized case previously presented, the difference highlights the impact of a semi-permeable membrane on the hydrodynamics of filtration. To draw a better comparison, we revert to the overall permeate flux and pressure drop as shown in Figs. 11 and 12. By increasing the alternating strand diameter ratio, R, from 1 to 1.5, the permeate flux per unit of pressure drop increased from 33 to 34.99 m^3^ and from 33 to 35.7 m^3^ for ratio R2. The percentage increase between R1 and R1.5 is 5.49% and between R1 and R2 is 7.627%. The pressure drop increase between R1 and R1.5 is 39.2% and between R1 and R2 is 72.9%.

### Length of wake region and vector plots

**Figure 13:**
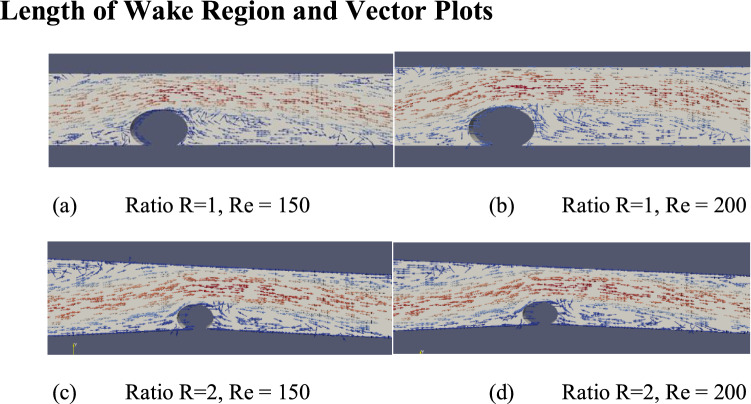
Vector plots of flow field around strands for strand ratio R of 1 & 2 and Reynolds numbers of 150 and 200.

The greater wake length combined with the added vortex strength furthers the cause of lesser polarization. The wake regions at different strand ratios and Reynolds numbers are shown in Fig. 13, while the average length of the wake region downstream of strands for the three-strand ratios is detailed in Table 2 below. The wake length is calculated up to the point where gradient flips along the lower wall of channel. The vector plots highlight the importance of strand ratio in the size and magnitude of the recirculation zone that increases mixing and reduces concentration polarization.

**Table 2:** Average magnitude of the wake region.

	R1	R1.5	R2
Wake length (cm)	19.777	20.250	20.253

Wall shear stresses, while following a similar trend, indicated stresses greater in magnitude through the length of the channel in higher Ratios. The stresses increased as the ratio increased.

## Conclusions

Spacer design in reverse osmosis extensively employs strands that have the same diameters, while they struggle to achieve greater permeate flux at lesser cost requires enhanced designs of strands. The alternate strand diameter offers an interesting solution. By reducing the strand diameter of the alternate strand, the impact of the turbulence promoters is further enhanced as the converging–diverging channel adds to the vorticity. For a minimal increase in pressure drop across the channel, greater vorticity can be achieved, which assists in the reduction of the concentration polarization.

To determine the maximum potential of a unit of the strand and by extension of the SWM, decoupled cases were prepared to focus on concentration studies in idealized scenarios and studies of membrane hydrodynamics. The latter focused primarily on determining the maximum permeate flux per unit of pressure drop across the unit. Concentration polarization retards the performance significantly. The decoupled flow ignores this aspect and allows the unit to perform at its full potential, thereby setting an ideal standard. This was followed by a study of the effects of different strand designs on concentration polarization reduction and combining the results with those of the membrane studies, the optimum strand design can be chosen for further investigation.

The studies concluded that a wavy channel provides greater permeate flux. It assists in preventing membrane fouling through added shear stresses and vorticity. The channel also creates a significantly higher transmembrane pressure differential.

## Data Availability

The datasets used and/or analysed during the current study available from the corresponding author on reasonable request.
